# In Vivo Detection and Measurement of Aortic Aneurysm and Dissection in Mouse Models Using Microcomputed Tomography with Contrast Agent

**DOI:** 10.1155/2019/5940301

**Published:** 2019-03-06

**Authors:** Guanglang Zhu, Huiying Sun, Jiannan Wang, Zhiqing Zhao, Junmin Bao, Rui Feng, Jian Zhou, Zaiping Jing

**Affiliations:** ^1^Department of Vascular Surgery, Changhai Hospital, Second Military Medical University, Shanghai, China; ^2^College of Animal Science and Technology, Anhui Agricultural University, Hefei, China

## Abstract

**Objectives:**

The aim of this study was to evaluate the potential of microcomputed tomography (micro-CT) using the intravascular contrast agent ExiTron nano 12000 for aorta imaging and monitoring the dynamic changing process of the aorta in mouse models with aortic aneurysm and dissection.

**Materials and Methods:**

Experiments were performed on healthy mice and mice with aortic dissection. Mice that were developing aortic dissection and healthy mice underwent micro-CT imaging after injection of ExiTron nano 12000. Time-dependent signal enhancement (at 1, 2, 3, 6, and 12 hours after intravenous injection of the contrast agent, respectively) in the aorta of healthy mice was measured to confirm the optimal imaging time of aorta. Various contrast agent doses (70, 100, and 150 *μ*l per 25 g mouse, respectively) were investigated to determine the optimal required dose for imaging of the aorta. The mice were scanned with micro-CT at 1, 14, and 28 days after onset of aneurysm and dissection to monitor the dynamic changing process of the aorta. Mouse aortas were stained with hematoxylin and eosin staining, and the diameter of the aorta was measured and compared with those obtained by micro-CT.

**Results:**

Time-dependent signal enhancement in the aorta shows that the contrast agent has a long blood half-life of 6 hours, with a peak enhancement at 2 hours after injection. Injection of 100 *μ*l ExiTron nano 12000 per 25 g mouse allows for effective visualization of the aorta. Micro-CT combined with contrast agent can monitor the changing process of the aorta in the mouse model of aortic aneurysm and dissection dynamically. The values of the diameter of the aortas obtained from the in vivo micro-CT imaging were compared with those obtained from histology and showed a significant correlation (*R*^2^ = 0.96).

**Conclusions:**

These data demonstrate that in vivo micro-CT is an accurate and feasible technique to detect aortic aneurysm or dissection in a mouse model, and the micro-CT technique using the innovative contrast agent ExiTron nano 12000 allows for monitoring various processes dynamically such as aortic remodeling in longitudinal studies.

## 1. Background

The incidence of aortic dissection (AD) in the world is 3 cases per 100,000 people per year [1]. Consequences of AD include the occlusion of blood flow to distant organs such as the brain, kidneys, bowels, and limbs; the formation of chronic aneurysms; rupture; or death [2–4]. At present, the biochemical and cellular processes involved in the initiation, development, and progression of AD are still poorly understood. Reproducible animal models of AD are key to understanding their pathophysiology. Mice are frequently used to create models for human diseases in the cardiovascular field because they can provide valuable information on the development and progression of cardiovascular diseases. In the previous study of AD, all of the mice had to be sacrificed, and only the initial and terminal states of the animals could be observed. The changes in the occurrence and development of AD and the changes in the prognosis of AD cannot be monitored dynamically. It is difficult to perform follow-up studies and pre- or postdisease development when someone wants to study the influence of drugs on disease development or prognosis.

Several noninvasive in vivo imaging techniques for mice have been developed including near-infrared fluorescence imaging [5], contrast-enhanced magnetic resonance imaging, positron emission tomography, ultrasound, and microcomputed tomography (micro-CT) [6–8]. The primary advantage of the micro-CT technique is high resolution which enabled identification of morphological changes in small structures. Further, noninvasive monitoring of pathological processes for human diseases in small animals is micro-CT, which has obtained more importance in the medical research in recent decades [9]. Noninvasive in vivo small animal micro-CT imaging provides excellent anatomical information at high resolution [10–12], but lacks sufficient soft tissues contrast [13]. To overcome the limited soft tissue contrast, the contrast agents were used to improve the differentiation of soft tissue compartments in vivo. To date, several preclinical contrast agents, ExiTron nano 6000, ExiTron nano 12000, Fenestra LC, Fenestra VC, eXIA 160, and eXIA 160XL, have been developed and are commercially available, which were applied to the liver and spleen imaging and cardiac imaging [14–18]. However, quite few research studies about micro-CT technique and contrast agents were applied to aortic aneurysm and dissection. From the previous study [15], we know that the contrast agent ExiTron nano 12000 is superior to the ventricle imaging when compared with other investigated contrast agents in terms of contrast agent accumulation over time. Therefore, we selected ExiTron nano 12000 for aorta imaging in our experiments.

In this study, we used the intravascular contrast agent ExiTron nano 12000, which has a high iodine concentration, to detect the dissected aorta and longitudinally monitor the changing process of the aorta in the mouse model of AD. Through measurement of the diameter of the aorta observed on the micro-CT scans and comparison with the values obtained from histopathology, the feasibility of the method to noninvasively monitor the changing process of the aorta and pathologic conditions in small animals was assessed.

## 2. Materials and Methods

### 2.1. Animals

All the experimental animal protocols were approved and reviewed by the Experimental Animal Center of Changhai Hospital in Shanghai, China. For our experiments, the healthy animal group consisted of three male C57BL/6 mice with a mean body weight of 22 ± 3 g. They were originally purchased from Charles River (Beijing, China) and housed at the animal care facility under specific pathogen-free conditions and fed a normal diet. For the AD group, we created AD in the indicated mice as previously described [19]. In brief, male mice at the age of three weeks on C57BL/6 backgrounds were administered *β*-aminopropionitrile monofumarate (BAPN) in drinking water at a dose of 1 g/kg/day (Sigma-Aldrich, St. Louis, MO, USA) for 4 weeks. Then, 1 *μ*g/kg/min angiotensin II (Ang II) was continuously infused subcutaneously for 72 hours using a micro-osmotic mini pump (Alzet, Cupertino, CA, USA). During micro-osmotic mini pump implantation, all the mice were anesthetized with 1.5% isoflurane.

### 2.2. Micro-CT Contrast Agents

The commercial micro-CT contrast agent used for mice studies in our experiment was ExiTron nano 12000 (Miltenyi Biotec, Bergisch-Gladbach, Germany). ExiTron nano is an alkaline earth metal-based nanoparticulate contrast agent, which is specifically formulated for preclinical CT imaging. The nanoparticles are totally stabilized by a polymer coating and have a mean hydrodynamic diameter of 110 nm. The contrast agent, ExiTron nano 12000, was used in the underlying study, and the densities of the undiluted contrast agents before injection were approximately 12,000 HU. To correct the contrast concentration in each animal, each animal was at about 25 g. Swabbing the tail with 70% alcohol before injection and then warming up the tail with warm water (40–45°C) provides better vessel dilation. The contrast agent was slowly injected intravenously via the lateral caudal vein. All tail vein injections were performed by a single experienced veterinarian.

To compare the elimination times of the contrast agents, three healthy male 8-week-old C57BL/6 mice were injected with the contrast agent ExiTron nano 12000 at a dose of 100 *μ*l per 25 g mouse. Micro-CT scanning was performed with an in vivo X-ray microtomograph scanner (Skyscan1076, Japan) at 1, 2, 3, 6, and 12 hours after intravenous injection of the contrast agent, respectively. At each time point, mean CT number (in Hounsfield units, HU) was measured in region of interest (ROI) within the aorta.

In order to determine the minimum required dose for imaging of the aorta, different contrast agent doses (70, 100, and 150 *μ*l per 25 g mouse, respectively) were investigated on three mice with C57BL/6 background. The dose-dependent signal enhancement was measured in ROI within the aorta at 2 hours after injection of ExiTron nano 12000. During this period, the mice were weighed and checked for their well-being every other day.

The mice were scanned with micro-CT at 1, 14, and 28 days after onset of aneurysm and dissection to observe the dynamic changing process of the aorta. At each time point, we measured the diameter of correspondingly segmental aorta with DICOM viewer.

### 2.3. Contrast-Enhanced Micro-CT Scanning Imaging

Micro-CT imaging was performed by in vivo X-ray microtomography scanner. Micro-CT imaging, enhanced using a contrast agent, was used to interrogate the location, the length, and the diameter of the dissected aorta in indicated animals. The animals were scanned at the indicated time points after intravenously injecting various doses of ExiTron nano 12000 to deeply anesthetized mice. After injecting the contrast agent, the animals were placed on the animal bed of the scanner. Before the micro-CT scanning was performed, a flat-field correction was taken to correct for variations in the pixel sensitivity of the camera. The system provides a detector with a matrix size of 908 × 600 pixels and a square pixel size of 36 *μ*m. The micro-CT acquisition parameters were 49 kV for X-ray tube voltage and 200 *μ*A for CT X-ray tube current with a field of view 20 mm and 360 degrees in one step. The acquisition time was 10 minutes. Images in HU were reconstructed in cubic voxels of 36.5 *μ*m, generating a matrix of 512 × 512 × 512 voxels. The CT data are converted to a DICOM format using the appropriate software. The gray value scaling is described as gray level center (*C*) and width (*W*) measured in HU, as commonly used in CT. In our study, two independent observers (one blinded) used a DICOM viewer to analyze the images to identify whether aortic aneurysm or dissection was present.

### 2.4. Image Analysis

The images obtained by micro-CT were analyzed by the public domain software DVStart. To make our results better comparable to other works, CT number (HU) was calculated from the images. Mean CT number was measured in ROI within the aorta. To compare the time course of contrast enhancement in the aorta, CT number within the aorta was analyzed. In addition, the diameter of the aorta was measured using DVStart software.

### 2.5. Histology

After micro-CT imaging, the seven mice with AD were immediately sacrificed, and the aorta of each mouse was removed. The sacrificed mice were perfused with phosphate-buffered saline (PBS) via the left ventricle to remove blood in the aorta. The entire aorta from the ascending aorta to the renal artery was excised and placed in sterile PBS, and then, the periadventitial fat was carefully removed. The location and scope of hematoma were carefully recorded by observing the aortic specimen. Subsequently, the mouse aortas were embedded in paraffin. Transverse slices of 10 *μ*m thickness were prepared, and mouse aortic tissues were stained with hematoxylin and eosin (H&E) staining. Photomicrographs were analyzed by investigators blinded to the experimental protocol with the aid of the NDP.view 2 software (Olympus) to assess the diameter of the aorta. In general, determination of the vascular lumen area was performed by planimetry of the lumen surrounded by tunica intima. In few cases, vessels became compressed during paraffin embedding. Therefore, the luminal diameter was always obtained from the luminal perimeter, assuming a circular shape. AD was confirmed by the H&E photograph, which was defined as the coexistence of true lumen and false lumen, even the thrombus in false lumen.

### 2.6. Statistical Analysis

The data are shown as means ± standard deviation (SD) or standard error of the mean (SEM). Correlations between micro-CT data and histological analysis were performed by linear regression. Statistical analyses were carried out in Graph Pad Prism 5. Values of *p* < 0.05 were considered statistically significant.

## 3. Results

All mice tolerated the injection of the various amounts of contrast agents well, and there were no adverse effects, such as abnormal behavior or sudden death.

**Figure 1:**
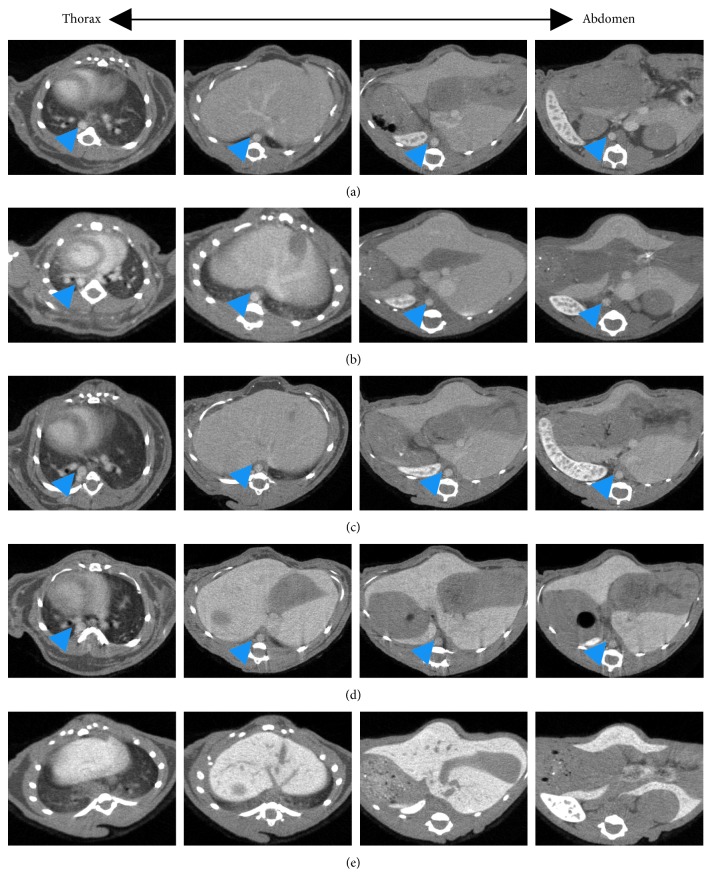
Time-dependent image of the aorta (arrowhead) from the proximal site of the descending thoracic aorta towards the abdominal aorta. Mice were subjected to serial contrast-enhanced computed tomography scanning at 1, 2, 3, 6, and 12 hours after intravenous injection of the contrast agent with 100 *μ*l per 25 g mouse, respectively.

**Figure 2:**
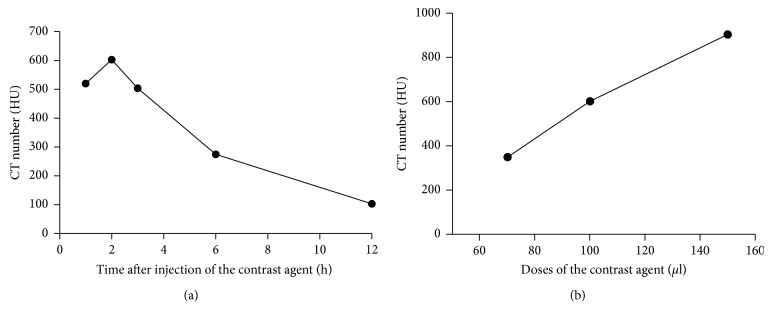
(a) Time course of contrast enhancement within the aorta of the three healthy C57BL/6 mice after a single tail vein injection of 100 *μ*l ExiTron nano 12000 per 25 g mouse. (b) Mean CT number (HU) at the second hour after a single tail vein injection of ExiTron nano 12000 at a dose of 75*μ*l/25g, 100*μ*l/25g, and 150*μ*l/25 g per mouse, respectively. Mean CT number was measured by placing a region of interest within the aorta.

To study the kinetics of contrast agent ExiTron nano 12000, time-dependent signal enhancement in the aorta of healthy mice was measured over a period of 12 hours after injection of ExiTron nano 12000 at a dose of 100 *μ*l per 25 g mouse.  Figure 1 shows representative CT images at 1 hour, 2 hours, 3 hours, 6 hours, and 12 hours postinjection. The contrast agent was still strong enough for imaging of the aorta at the sixth hour after injection of ExiTron nano 12000. The CT images show that the contrast agent has a long blood half-life of approximately 6 hours, with a peak enhancement observed at the second hour after injection. At 12 hours postinjection, the signal enhancement in the aorta has nearly reached a baseline value and was insufficient for the aorta imaging, showing that at this time point, the contrast agent has been practically cleared from the blood. The average CT number for the three mice at each time point is plotted in Figure 2(a). The optimal aorta contrast enhancement was observed approximately at the second hour after a tail vein injection of 100 *μ*l ExiTron nano 12000 per 25 g mouse. ExiTron nano 12000, which was injected only once before the first micro-CT scanning was performed, similarly induced contrast of the aorta that decreased slightly over time. In particular, we could detect the AD with the micro-CT, and the aorta is easily differentiated from the surrounding structures at both time points. Because imaging at 2 hours after injection of ExiTron nano 12000 allows for effective visualization of the aorta, this time point could be selected for subsequent studies in mouse models of AD.

**Figure 3:**
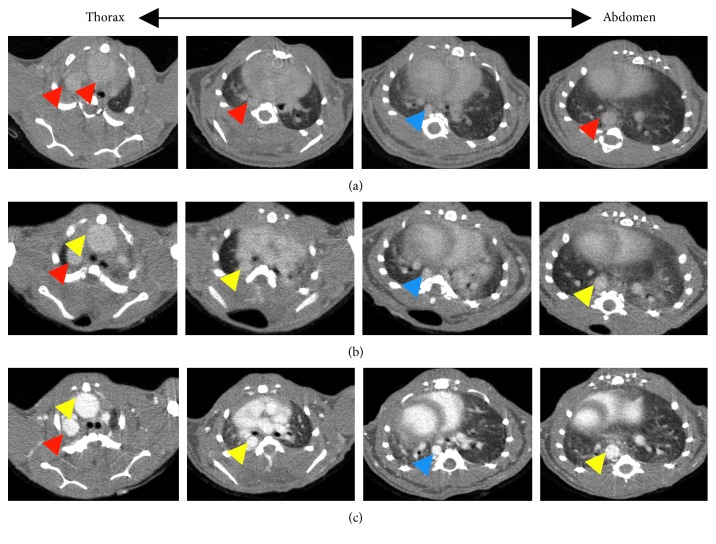
Contrast agent doses dependent for the image of the aorta 2 hours after injection of ExiTron nano 12000 at a dose of 75 *μ*l/25 g, 100 *μ*l/25 g, and 150 *μ*l/25 g per mouse, respectively. The images represent the aortic aneurysm and dissection imaging with different contrast agent doses from the proximal site of the descending thoracic aorta towards the abdominal aorta. Yellow, red, and blue arrowheads indicate the dissected aorta, the dilated aorta, and the normal aorta, respectively.

**Figure 4:**
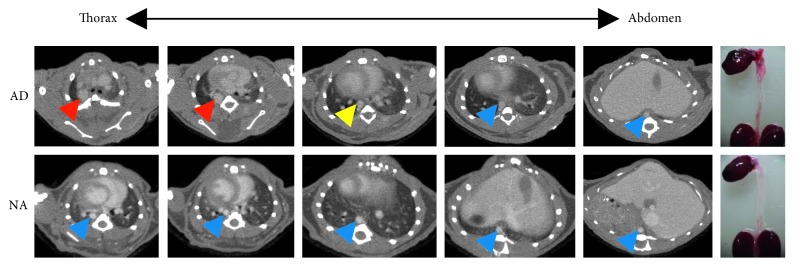
Micro-CT image of a mouse with AD and NA 2 hours after injection of ExiTron nano 12000 at a dose of 100 *μ*l/25 g mouse. Yellow, red, and blue arrowheads indicate dissected aorta, dilated aorta, and NA, respectively. NA, normal aorta.

Various contrast agent doses were investigated to determine the minimum required dose for imaging of the aorta. Different contrast agent doses, 70 *μ*l, 100 *μ*l, and 150 *μ*l ExiTron nano 12000 per 25 g mouse, were investigated on the C57BL/6 mice with AD. AD could be detected with 100 *μ*l and 150 *μ*l ExiTron nano 12000 per 25 g mouse (Figure 3). As expected, the signal enhancement in the aorta is increasing with the amount of contrast agent injected, and the result is shown in Figure 2(b). From the previous study, we know that the ExiTron nano 12000 could induce toxicological side effects on the body weight, thyroxine, total protein, and blood counts [15]. These toxicological side effects could affect the animal models and increase the uncontrollable factors in the experiment, which would not happen without the contrast agents. Therefore, we must use as small of amount of contrast agents as possible in our experiment under the premise that AD could be detected. Nevertheless, the AD imaging could be detected using ExiTron nano 12000 at a dose as low as 100 *μ*l per 25 g mouse, which is particularly beneficial for the weak and diseased small animals with severe aortic disease. As observed in Figure 4, imaging at dose of 100 *μ*l ExiTron nano 12000 per 25 g mouse in the aorta signal enhancement at 2 hours postinjection, which is sufficient for aortic morphological studies. These results showed that 100 *μ*l ExiTron nano 12000 per 25 g mouse could be used in our following experiments. Imaging after injection of 100 *μ*l ExiTron nano 12000 per 25 g mouse allows for effective visualization of the aorta; so, this volume of ExiTron nano 12000 was selected for subsequent studies in mouse models of AD.

**Figure 5:**
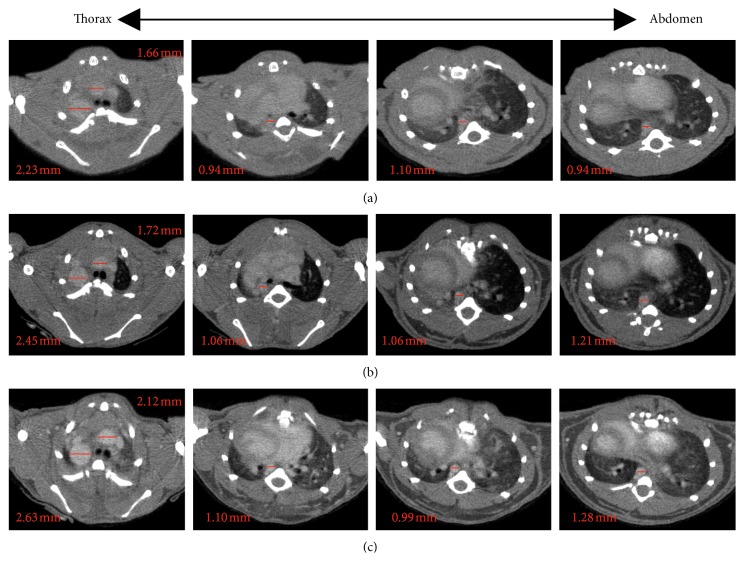
Time-dependent dynamic changing process of the aorta in dissected mice visualized by serial contrast-enhanced CT scanning at the 1st, 14th, and 28th day after the AD on set with the injection of ExiTron nano 12000 at a dose of 100 *μ*l/25 g mouse.

Micro-CT combined with ExiTron nano 12000 was performed at the 1st day, the 14th day, and the 28th day after dissection on set to observe the dynamic changes of the aorta. Time course examination of serial CT imaging, enhanced with the contrast agent, showed that AD invariably emerged in BAPN/Ang II-treated mice at the 1st day after dissection on set. It initiated at the proximal site of the descending thoracic aorta. The diameter of the dissected thoracic aorta has been changed at the 14th day and the 28th day and could be observed by micro-CT (Figure 5).

**Figure 6:**
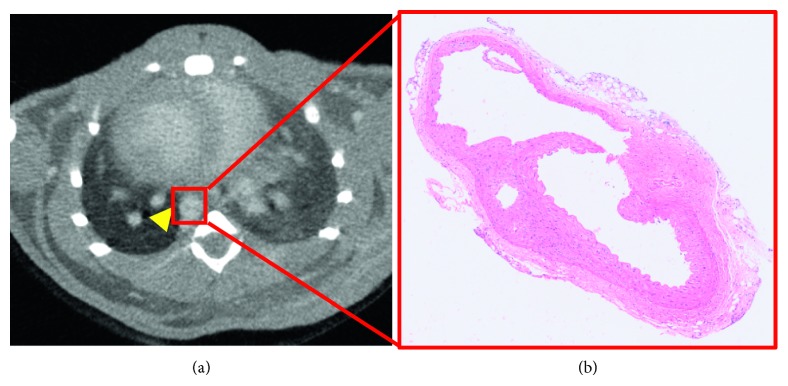
Micro-CT image of mouse with AD 2 hours after injection of ExiTron nano 12000 at a dose of 100 *μ*l/25 g mouse (a) and corresponding microscopy image of histological section (b). Yellow arrowhead indicates dissected aorta.

**Figure 7:**
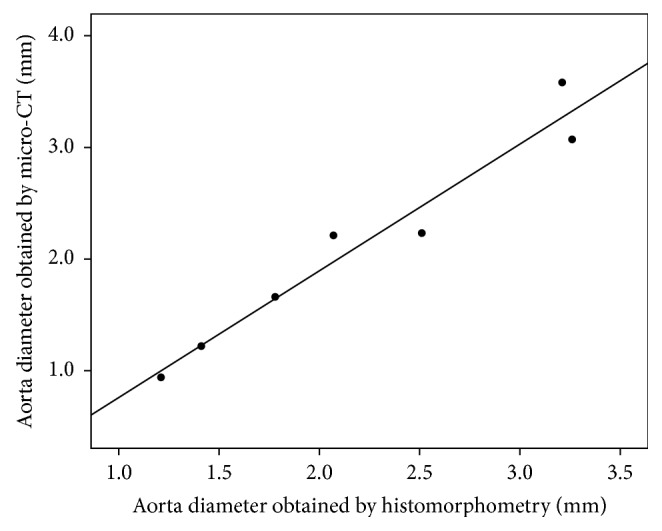
Regression plots for the diameter of the aorta obtained via micro-CT imaging and by histomorphometry, showing a significantly strong correlation (*R*^2^ = 0.96).

To access the effect of micro-CT on the measurement of AD in mice, signal enhancement in the mice with AD (*n*=7) at 2 hours after injection of the contrast agent at a dose of 100 *μ*l per 25 g mouse was used for subsequent measurement of the diameter of the aorta. Through micro-CT images, the diameter of the aorta was measured and compared with those obtained by histopathology. A qualitative comparison of the micro-CT images with microscopy images of histological sections indicated that the contrast agent is homogeneously distributed in the aorta and that AD could be detected by micro-CT with ExiTron nano 12000 (Figure 6). The regression of the data is shown, and a quantitative comparison of the diameter of the aorta obtained via the two methods showed a significantly strong correlation (*R*^2^ = 0.96) (Figure 7). This demonstrates that micro-CT using ExiTron nano 12000 allows for noninvasive studies of AD or aneurysm without the need of sacrificing the small animals. A paired *t*-test for equality of the micro-CT and histopathology measurements could find no statistically significant difference between the two techniques (*t*=−0.842 and *p*=0.432).

## 4. Discussion

Mouse models are currently used to study pathological processes of cardiovascular diseases and the efficacy of novel therapeutic regimens. All of the mice in the experiments had to be sacrificed, and only the initial and terminal states of the small animals could be observed in the previous study of AD. The researchers had difficulty monitoring the changes in the occurrence and development of AD or the changes in the prognosis of AD dynamically. It is challenging to understand the influence of drugs on disease development or prognosis. To our knowledge, there are quite few studies about the in vivo detection of AD in mouse models using micro-CT with contrast agents. Trachet et al. used micro-CT and high-frequency ultrasound combined with phase-contrast X-ray tomographic microscopy (PCXTM) and PCXTM-guided histology to obtain a detailed insight into the global 3D morphology at different locations of dissecting aortic aneurysm [20, 21]. They did not elaborate on the details of aorta imaging in mice using micro-CT and contrast agent. In our study, we longitudinally monitored the dissecting aorta in vivo at different time points using micro-CT combined with contrast agent. Micro-CT allows noninvasive and dynamic detection of highly detailed vascular pathologic conditions, monitoring these vascular processes in the live mice models at high resolutions. Micro-CT provides detailed imaging of vascular structures, which can help in obtaining an understanding of the underlying angiogenesis and vascular biology. The changes of the vascular system can be observed dynamically. In this technique, in vivo micro-CT mainly depends on the contrast agent to increase the resolution. The lumen of a blood vessel enhanced can be differentiated from those that are nonenhanced soft tissues by the use of intravascular contrast-enhanced media. ExiTron nano 6000, ExiTron nano 12000, Fenestra LC, Fenestra VC, eXIA 160, and eXIA 160XL are six such intravascular contrast agents that are available for micro-CT. The contrast enhancement in the left ventricle, liver, spleen, kidney, and muscle with these six intravascular contrast agents have been reported immediately following the injection, 3 hours and 120 hours postinjection [15]. With ExiTron nano 12000 being for preclinical use only, it was not applied for clinical use in the current situation. Rothe et al. [22] and Mannheim et al. [15] reported that ExiTron nano 12000 is superior for heart imaging compared with other contrast agents and yielded the strongest contrast enhancement in the blood pool. The contrast agents eXIA 160 and 160XL, Fenestra VC and LC showed a contrast enhancement just shortly after injection. This is most likely a result of the contrast agent chosen for AD imaging in our study.

The injection of larger volumes of contrast agent can be problematic, especially in small animals such as mice, which have a total blood volume in the range of 2.0 to 3.0 ml. Willekens et al. [23] reported that the mice were prone to die if the injected volume is greater than 0.2 ml/20 g. Compared with other contrast agents such as eXIA and Fenestra, ExiTron nano provides similar or significantly stronger contrast enhancement in the left ventricle, liver, and spleen at considerably lower injection volumes [15]. The reduced injection volume of ExiTron nano is facilitated by the high concentration of the alkaline earth metal, which is possible owing to the contrast agents' nanoparticulate formulation. From our experience, in most cases, ExiTron nano 12000 provides sufficient contrast for imaging of the aorta with 100 *μ*l per 25 g mouse.

In our article, the contrast agent distribution was determined in healthy male C57BL/6 mice. Suckow and Stout. reported that the uptake of the contrast agents in the spleen was higher in severe combined immunodeficient (SCID) mice than the other strains and that the highest uptake was found in the liver of C57BL/6 mice compared with C57BL/6 and SCID mice [24]. These results demonstrated that the pharmacokinetics may be distinguished in different mouse strains, and the aorta imaging may be better in other mouse strains. The most commonly used mice in cardiovascular research are C57BL/6 mice, so we use C57BL/6 mice in our research rather than SCID mice.

The ability of micro-CT to accurately detect and quantify AD is an important step for preclinical imaging in animal models. Micro-CT has been widely used in preclinical imaging for the evaluation of a variety of vascular-related therapeutics because of its high spatial resolution. ExiTron nano 12000 improves the usefulness of micro-CT by providing higher enhancement for aorta imaging with a contrast agent. ExiTron nano 12000 is also an improvement over the delayed enhancement seen in clinical CT for aorta imaging, using traditional iodinated contrast agents.

In summary, the protocol described in the article provides a safe technique for the high-resolution detection of the vasculature of a mouse model with AD. Quantitative analyses can be easily achieved by micro-CT imaging with ExiTron nano 12000.

Contrast-enhanced micro-CT imaging is a promising imaging modality for the study of vascular biology and enables the assessment of the vascular complications noninvasively and in vivo. The procedure described in our article requires multiple skills and techniques, including animal handling and preparation, image acquisition, and quantification of the imaging results. It is a promising imaging modality for the study of vascular biology. Further studies are needed to assess its applicability in other animal models and disease processes.

## 5. Conclusion

Micro-CT using the innovative contrast agent ExiTron nano 12000 offers an opportunity to evaluate the pathology and structure of blood vessels with high spatial resolution. Our present study demonstrates that in vivo micro-CT is equally suited to histology for the determination of the vessel lumen. In addition, micro-CT combined with the innovative contrast agent ExiTron nano 12000 could be developed to in vivo monitor the dynamic changing process of AD such as AD remolding in small animals.

## Data Availability

All the continuous data and image data used to support the findings of this study are included within the article.
